# Comparison of the Physical Care Burden on Formal Caregivers between Manual Human Care Using a Paper Diaper and Robot-Aided Care in Excretion Care

**DOI:** 10.3390/ijerph20021281

**Published:** 2023-01-10

**Authors:** Jeong-Bae Ko, Yong-Ku Kong, Kyeong-Hee Choi, Chang-Ki Lee, Hyun-Ji Keum, Jae-Soo Hong, Byeong-Hee Won

**Affiliations:** 1 Digital Healthcare R&D Department, Korea Institute of Industrial Technology, Cheonan 31056, Chungcheongnam-do, Republic of Korea; 2 Department of Industrial Engineering, Sungkyunkwan University, Suwon 16419, Gyeonggi-do, Republic of Korea

**Keywords:** excretion care, formal caregiver, physical care burden, manual human care, robot-aided care

## Abstract

Although the older population has been rapidly growing, the availability of formal caregivers remains limited. Assistance provided by care robots has helped lower this burden; however, whether using a care robot while providing excretion care (EC) is quantitatively increasing or decreasing caregivers’ physical care burden has not been extensively studied. This study aimed to quantitatively compare the physical burden experienced by caregivers while providing manual excretion care (MC) using a paper diaper versus robot-aided care (RC). Ten formal caregivers voluntarily participated in the experiment. MC and RC tasks were structuralized according to phases and classified by characteristics. The experiment was conducted in a smart care space. The physical load of formal caregivers was estimated by muscular activity and subjective rating of perceived physical discomfort. The results demonstrated that although the physical load on the lower back and upper extremities during the preparation and post-care phases were greater in RC than MC, RC markedly alleviated caregivers’ physical load when performing front tasks. In the preparation-care phases, the physical loads on the lower back and upper extremities were approximately 40.2 and 39.6% higher in the case of RC than MC, respectively. Similar to the preparation-care phases, the physical loads on the lower back and upper extremities during post-care phases were approximately 39.5 and 61.7% greater in the case of RC than MC, respectively. On the other hand, in the front-care phases, the physical loads on the lower back and upper extremities were approximately 25.6 and 34.9% lower in the case of RC than MC, respectively. These findings can quantitatively explain the effectiveness and features of a care robot to stakeholders and provide foundational research data for the development of EC robots. This study emphasizes the implementation and promotion of the dissemination, popularization, and development of care robots to fulfill formal caregiving needs.

## 1. Introduction

The demand for formal caregivers of older adults has been growing with the rapidly increasing global aging population. South Korea is anticipated to become a super-aged society in 2025; those aged 65 years and over are expected to exceed 10 million and account for more than 20% of the total population. Additionally, approximately 21% of the older population may require caregiving [1]. By 2030, a total of 750,000 formal caregivers will be required, but the number is projected to fall short by 110,000 [2]. The shortage of caregivers can lead to an increase in care costs for the elderly. The care costs accounted for 0.335% of South Korea’s total GDP as of 2020 but are expected to increase to 1.856% in 2060 [3]. The care receivers and their families will suffer from financial burdens as the increase in care costs. This is also expected to increase the workload of available caregivers and produce adverse effects, such as stress, musculoskeletal disorders, and impaired quality of life [4,5]. Such adverse effects can lead to the abuse or neglect of older adults [6].

Caregivers assist older adults with activities of daily living (ADLs), such as toileting, bathing, and transferring, which account for approximately 80% of their work. Providing repetitive assistance with ADLs may be physically straining for caregivers. Specifically, providing excretion care (EC) using a paper diaper is considered physically demanding, as care receivers require repeated assistance in this context. For instance, according to Darragh et al. [7], caregivers had to change the diapers of every senior citizen admitted to nursing homes an average of four to six times per day; as a result, most caregivers suffered from musculoskeletal pain and physical fatigue. Additionally, providing EC involves repositioning the body of the care receiver; therefore, caregivers providing repeated EC can potentially have work-related muscular musculoskeletal disorders (WMSD) [8,9,10,11].

With the advancement of robot technologies, further research is being conducted, and various types of care robots assisting care receivers with their ADLs are being developed to lower caregivers’ physical burden. Yeom et al. [12] designed a medical bed for a bedridden patient, which can automatically detect, transport, and store urine and feces in bed, and they expected care receivers to decrease their dependency on caregivers using the medical bed. However, the medical bed was not a commercial product but a prototype. Park et al. [13] presented a new meal-assistance robot for people with motor impairments, which can track a human’s mouth pose based on vision. The experimental results demonstrated the safety and usability for the user, and it helps people with motor impairments independently eat meals. An assistive bathing robot (I-Support) developed by Zlatintsi et al. can detect human activity and support the elderly and caregivers to transfer into a bathing cabin [14]. Chen et al. [15] developed a deep-learning model to estimate the human joint position for a transfer robot. When the model was applied to the transfer robot, the accuracy for recognition of human joints was higher than before. Zhao et al. [16] developed an auxiliary lavatory robot with autonomous movement capability and a comparatively convenient cleaning method for supporting EC. As the experimental results show, the auxiliary lavatory robot could improve the safety and convenience of the user and reduce the physical load of caregivers.

As mentioned above, some care robots can now assist with providing EC. Lee et al. [17] conducted a questionnaire survey on nurses participating in care practices with assistance from care robots and found that EC is one of the highest-ranked care types in this context. The study indicated that a care robot assisting with EC could reduce nurses’ workload and enhance the quality of provided nursing care. Robots assisting with EC were categorized into three types depending on their purpose: those providing assistance with (1) disposal of excrement, (2) prediction of excretion, and (3) maintaining excretion posture [18,19]. The first type of robot automatically disposed of the excreted waste and assisted bedridden care receivers in performing their excretion activity in their rooms [12,20]. The second type sent an alert to the care receivers when their bladder was full as a reminder for them to go to the restroom; this type of robot was designed to help older adults who are capable of performing such an ADL on their own but require assistance because of their impaired cognition or incontinence [21]. Finally, the third considered type of robot helped care receivers perform body movements, such as undressing and sitting on a toilet, which care receivers need to perform before using the restroom [16,22].

Prior to this study, we conducted a focus group interview with 73 formal caregivers and long-term care (LTC) facility staff to discuss the use and implementation of EC robots in nursing homes. They had positive opinions about the implementation of robots assisting with EC in the presence of relevant supportive policies (65%). One negative opinion received was that EC robots actually increase the workload and are not user-friendly, even though they were developed to alleviate the care burden (42%). According to Na et al. [23], when the authors conducted face-to-face interviews with 13 care workers who hadn’t experienced the EC robots, most care workers were concerned that using the EC robots would make the situation only more complicated. The authors described the causes of these responses as care workers had the perception that care robots would not respond as skillfully as humans in EC. Regarding the negative opinion, we investigated how much the usage of care robots for EC quantitatively increased or decreased caregivers’ physical care burden; however, limited has been conducted in this regard. Homma et al. [24] considered muscular activities to assess the physical burden placed on care receivers by robots assisting with EC. They found that muscular activity in the lower back and knee was lower during excretion when they used a care robot than when they did not. However, their study only assessed the reduction of care receivers’ physical burden and not the alleviation of caregivers’ physical burden.

Therefore, this study aimed to quantitatively analyze caregivers’ physical burden when providing robot-aided care (RC) in the context of EC; we further compared this physical burden with that experienced while providing manual excretion care (MC) using a paper diaper. The physical burden was quantitatively assessed based on measured muscular activities using a surface electromyography measurement system (sEMG); a subjective rating of perceived physical discomfort was obtained to compare the subjective physical burdens recorded in the context of MC and RC.

## 2. Materials and Methods

### 2.1. Participants

This study recruited 10 formal caregivers to compare their care burden experienced while providing MC and RC. The inclusion criteria included having a caregiver certificate, working as a formal caregiver in an LTC facility or providing in-home care services, not having a history of musculoskeletal disorders, and having the ability to comprehend and independently follow the instructions provided during the study. The participants agreed to participate in the study voluntarily and provided informed consent. All the participants were female, with a mean age of 61.7 ± 11.3 years and a mean career length of 3.3 ± 6.7 years.

### 2.2. Experiment

#### 2.2.1. Apparatus

**Figure 1:**
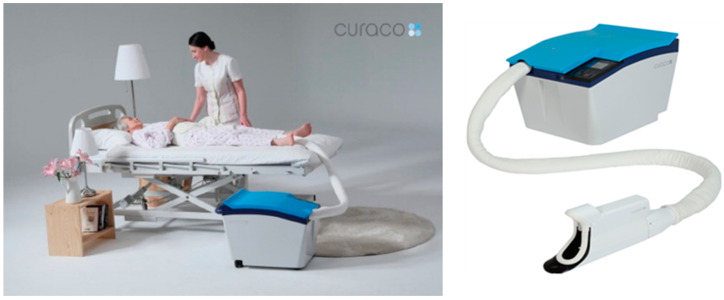
The excretion care robots: CareBidet. Reprinted with permission from Ref. [20]. 2018, CURACO.

EC is performed differently depending on the state of care receivers [23]. Some care receivers are able to move independently to the toilet but have difficulty disposing of excrement. In the case of these care receivers, caregivers help the care receiver to keep their body balanced for the disposal of excrement. Other care receivers can be bedridden. Bedridden care receivers need caregiving which washes the excrement on the anus and changes a Foley catheter or a paper diaper. When caregivers perform EC for bedridden care receivers, caregivers turn the body of bedridden care receivers. Frequently turning care receivers can be a cause of WMSD for caregivers [25]. Bedridden care receivers also need supportive EC at night, which can accumulate the workload for caregivers. EC for bedridden care receivers is more intensive than other types of EC care receivers. Therefore, we focused on EC for bedridden care receivers. This study used the care robot CareBidet (CURACO, Seongnam, Republic of Korea) to assist with EC (Figure 1). It automatically detects the excrement, removes it through suction, and disposes of it. Moreover, it can wash and dry the area of the anus afterward. It comprises two units: the wearing unit, which is directly connected to the care receiver, detects the excrement, and rinses the anus of excretion; the main body, which collects the excrement through suction.

Although the scope of using CareBidet is limited to bedridden care receivers, it can lower caregivers’ workload and alleviate their physical care burden. The specifications of CareBidet are illustrated in Table 1.

#### 2.2.2. Experimental Task

The structure of providing MC was based on the Care Task guidelines for EC, published by the National Health Insurance Service (NHIS) [26]. Following a review of the designed MC plan by experts, we excluded the tasks that are not routinely performed in nursing homes, such as the regular education for formal caregivers and evaluation of the care ability. The finalized MC plan comprised 72 tasks. The plan for RC was structured following the user guidelines provided by CareBidet and was restructured based on the care manager education plan provided by CURACO. Tasks that are not routinely performed, such as hose disinfection and drainage management, were not considered in the experiment. The RC plan comprised 130 tasks. The details of the MC and RC tasks are discussed in Appendix A.

#### 2.2.3. Experimental Procedure

**Figure 2:**
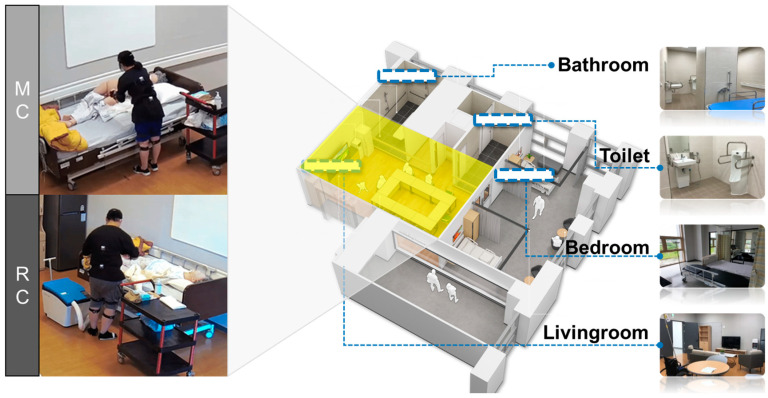
The smart care space simulates a nursing home.

The experiment was conducted in a smart care space (see Figure 2) designed to resemble an actual nursing home. It included an observation room where the operator could observe the participants without disrupting the experimental flow and a video recording system that recorded the participants’ behaviors during the experiment. We recorded the participants’ MC and RC tasks using this system and used the recordings to measure the duration of the tasks.

**Figure 3:**
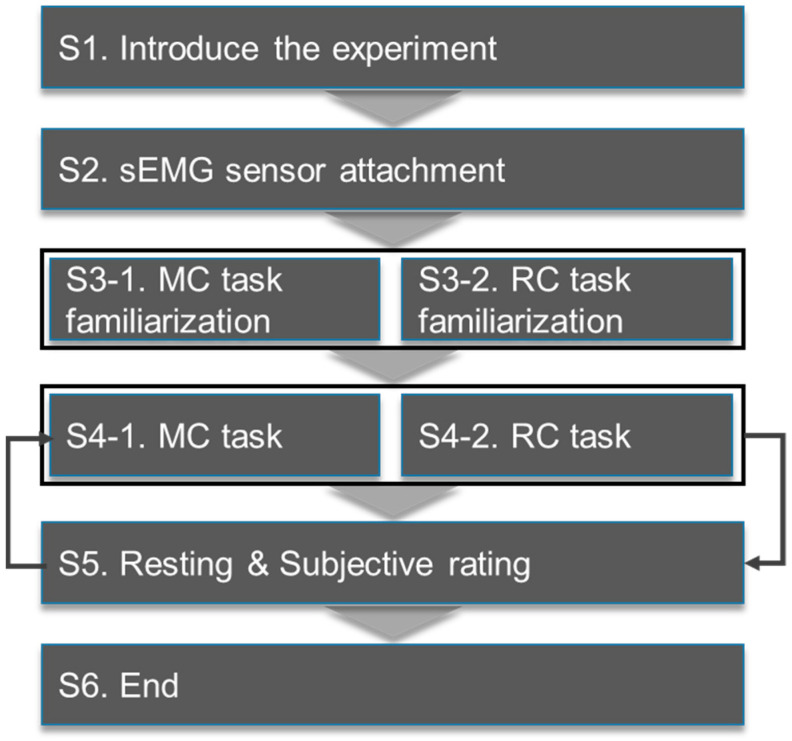
The experimental procedure.

The experimental flow chart is illustrated in Figure 3. Before the experiment, the objectives and procedure of the experiment were explained to participants, following which they signed a consent form. The participants specified their handedness, and eight sEMG sensors were attached based on their dominant hand. Nine out of ten participants were right-handed, and one was left-handed. After the attachment of the sEMG sensors, the maximum voluntary contraction (MVC) of eight muscles was measured for 5 s. The MVC for each muscle was measured using the MVC measurement guideline from the surface EMG to ensure the non-invasive assessment of muscles [27]. Before data collection, the participants were adequately educated and trained in the MC and RC tasks to ensure that they could independently perform them during the experiment. A dummy, which simulated an older female, was used as the role of a care receiver. The dummy weighed about 45 kg and was 156 cm tall. MC and RC were performed in the order of an actual EC. After the completion of each task group, the participants were asked to provide a subjective rating of perceived physical discomfort.

### 2.3. Measurement and Data Analysis

#### 2.3.1. Hierarchical Task Grouping

**Figure 4:**
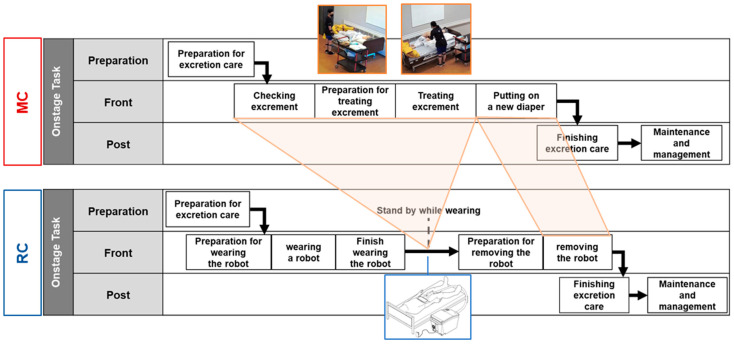
The structure of the hierarchical excretion care task (upper figure: manual care using a paper diaper; lower figure: robot-aided excretion care).

MC and RC cannot be directly compared, as their sub-tasks differ. The tasks were structured and classified into a time series to compare the physical burden for MC and RC. First, the care tasks were grouped into three categories; (1) Preparation task: tasks involved in preparation for MC and RC, such as the preparation of supplies or checking for excretion; (2) Front task: actual performance of MC and RC, such as changing of the diaper, washing of the perineal area, or accoutering of the wearing unit; (3) Post task: tasks that conclude the care, such as the tidying of the area, or storing of supplies. Subsequently, the tasks in each category were clustered into groups on the basis of their order. Figure 4 illustrates the hierarchical task grouping for MC and RC.

#### 2.3.2. Muscular Activity

**Figure 5:**
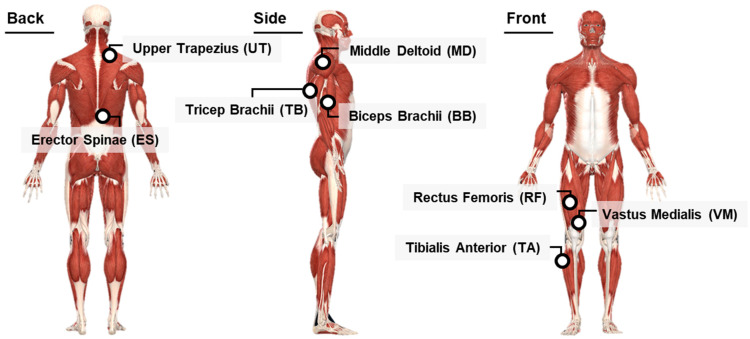
Positions of eight sEMG sensors.

The Trigno wireless EMG system (Delsys, Natick, MA, USA) was used to measure the muscular activities of formal caregivers during MC and RC tasks. The muscles in the lower back, cervical vertebrae, shoulder, upper extremity, thigh, and shin were selected as representative muscles of the body parts where formal caregivers often experience physical discomfort and pain due to repetitive EC work [7,28]. As illustrated in Figure 5, eight sEMG sensors were attached: Erector Spinae (ES), Upper Trapezius (UT), Middle Deltoid (MD), Tricep Brachii (TB), Biceps Brachii (BB), Rectus Femoris (RF), Vastus Medialis (VM), and Tibialis Anterior (TA). The sEMG sensors used for the experiment were embedded in an electrode and attached to eight muscles following the SENIAM guidelines. Muscular activity data were analyzed using the EMGworks software 4.8.0 (Delsys, Natick, MA, USA). EMG signals were sampled at 2100 Hz and processed through a Butterworth second-order band pass filter (low frequency of 20 Hz; high frequency of 450 Hz) [29]. The root means square (RMS) of the five-second period of each MVC was computed from collected electrical muscle signals (μV). The RMS values for each collected electrical muscle signal during the experiment were normalized as the RMS value for the five-second period of each MVC, presented as %MVC. The %MVC was calculated using the following equation [30]:

(1) $$\%\mathrm{MVC}=\left[\frac{EMG_{task}-EMG_{resting}}{EMG_{MVC}-EMG_{resting}}\right]\times 100\;\left(\%\right)$$

*EMG_task_* is the value measured during a task, and *EMG_MVC_* is a value measured during MVC measurement. *EMG_resting_* is a value measured during rest before MVC measurement. The %MVC for eight muscles was calculated for each task.

#### 2.3.3. Subjective Rating of Perceived Physical Discomfort

The subjective rating of perceived physical discomfort was assessed using the Borg RPE (ratings of perceived exertion) scale [31]. This scale measures one’s effort, exertion, breathlessness, and fatigue during physical work and is highly associated with occupational health [32]. The Borg RPE scale rates each type of physical work on a scale of 6–20 and is highly correlated with heart rate. For example, if someone rates a particular task as six, the intensity of the task is at approximately 60 bpm, which thereby suggests an easy task. In contrast, if someone rates a particular task at twenty, it suggests that the task is particularly straining at approximately 200 bpm. This study collected RPE scores for each task in MC and RC, and the %RPE was calculated via minimum-maximum normalization using the following equation:

(2) $$\%\mathrm{RPE}=\left[\frac{RPE_{task}-RPE_{min}}{RPE_{max}-RPE_{min}}\right]\times 100\;\left(\%\right)$$

*RPE_task_* is the RPE score for each task. *RPE_min_* is the minimum RPE score of 6, and *RPE_max_* is the maximum RPE score of 20. The %RPE was used to compare the subjective physical discomfort between MC and RC.

### 2.4. Statistics

This study’s independent variables included MC and RC tasks; its dependent variables included the muscular activity of eight muscles and the subjective physical discomfort for each task. The normality of the muscular activity data and subjective physical discomfort data for each MC and RC task was tested at a significance level of 95% using the Shapiro–Wilk test. The results indicated that none of the collected data were normally distributed (*p* < 0.05). Thus, muscular activities and subjective physical discomfort between MC and RC were compared using a non-parametric method, namely the Wilcoxon rank-sum test.

## 3. Results

### 3.1. Muscular Activity

**Figure 6:**
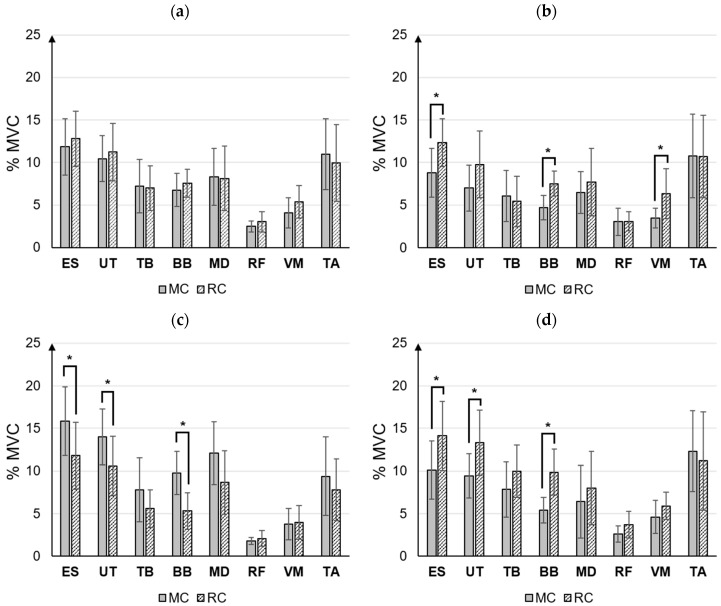
The comparison of muscular activity between MC and RC: (**a**) Total MC vs. Total RC, (**b**) Preparation MC vs. Preparation RC, (**c**) Front MC vs. Front RC, and (**d**) Post MC vs. Post RC. Note * *p* < 0.05.

There were no statistically significant differences in the muscular activities of the eight muscles when comparing all tasks between MC and RC (Figure 6a). However, when comparing hierarchical task groups in the Preparation, Front, and Post categories between MC and RC, a few muscles demonstrated significant differences in muscular activity.

The muscular activities of ES, BB, and VM demonstrated significant differences between MC and RC in the Preparation task (*p* < 0.05). The muscular activities of the ES, BB, and VM were all higher in RC than in MC (Figure 6b). ES was 40.2% higher in RC (12.3 ± 2.8%) than in MC (8.8 ± 2.8%), and BB was 39.6% higher in RC (7.5 ± 1.5%) than in MC (4.7 ± 1.4%). VM was 81.6% higher in RC (6.3 ± 2.9%) than in MC (3.5 ± 1.1%).

In the Front task, the muscular activities of ES, UT, and BB significantly differed between MC and RC (*p* < 0.05). ES, UT, and BB reported lower muscular activity in RC than in MC (Figure 6c). ES was 25.6% lower in RC (11.8 ± 3.8%) than in MC (15.8 ± 4.0%), and UT was 24.3% lower in RC (10.6 ± 3.5%) than in MC (14.0 ± 3.7%). The muscular activity of BB was 45.5% lower in RC (5.3 ± 2.2%) than in MC (9.8 ± 2.5%).

In the Post task, the muscular activities of ES, UT, and BB significantly differed between MC and RC (*p* < 0.05). The muscular activities of ES, UT, and BB were higher in RC than in MC (Figure 6d). The muscular activity of ES was 39.5% higher in RC (14.1 ± 4.1%) than in MC (10.1 ± 3.4%), and UT was 41.3% higher in RC (13.3 ± 3.8%) than in MC (9.4 ± 2.6%). BB muscle activity was 82.1% higher in RC (9.9 ± 2.7%) than in MC (5.4 ± 1.5%).

### 3.2. Subjective Rating of Perceived Physical Discomfort

**Figure 7:**
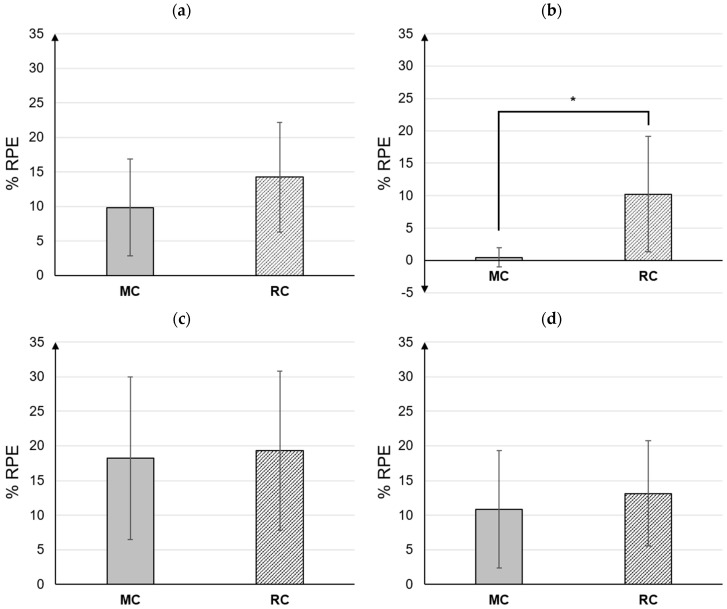
The comparison of subjective physical discomfort between MC and RC: (**a**) Total MC vs. Total RC, (**b**) Preparation MC vs. Preparation RC, (**c**) Front MC vs. Front RC, and (**d**) Post MC vs. Post RC. Note * *p* < 0.05.

Subjective physical discomfort during all MC and RC tasks was compared; however, there were no significant differences between the two groups (Figure 7a). Regarding hierarchical task grouping, there were statistically significant differences between MC and RC only during the Preparation task (*p* < 0.05). During this phase, subjective physical comfort was higher in RC (10.2 ± 8.9%) than in MC (0.5 ± 1.5%) (Figure 7b). Figure 7c,d illustrates the subjective physical discomfort during the Front and Post tasks.

## 4. Discussion

This study aimed to compare the physical care burden experienced by formal caregivers in the context of MC and RC using muscular activity and subjective rating of perceived physical discomfort. First, we specified the MC and RC tasks based on the previous literature, conducted a focus group interview, and verified the tasks suggested by experts. Subsequently, we recruited ten formal caregivers who performed the MC and RC tasks according to their sequence, measured their muscular activity, and observed subjective physical discomfort. Finally, we compared the %MVC and %RPE between MC and RC and confirmed which phases (Preparation task, Front task, and Post task) of MC and RC demonstrated high care burden; we also determined the EC methods that were more effective in terms of the alleviation of the physical care burden.

The muscular activities of eight muscles observed in the context of MC and RC were compared based on the hierarchical care task group. Although no significant differences in muscular activities were observed with regard to the total number of tasks, muscular activities of the upper body muscles, namely ES, UT, and BB, differed with regard to the Preparation, Front, and Post tasks. Specifically, ES and BB differed in all task phases, and it was found that these muscles are commonly affected during caregivers’ repeated performance of EC. Davis and Kotowski [33] reported that 65% of formal caregivers who work in hospitals, long-term care facilities, and home healthcare settings experienced lower back injuries after providing assistance with repetitive body positioning activities, and 54% of them experienced a shoulder injury.

In the Preparation and Post tasks, ES, UT, and BB demonstrated higher muscular activity in RC than in MC. Furthermore, RC demonstrated higher subjective physical discomfort in the Preparation task compared with MC. These results suggest that bringing the robot, installing it at the bedside for RC, washing the waste container, and storing the robot may induce physical load on the lower back and upper extremities. Additionally, RC required a greater number of tasks than MC, and the caregivers were not accustomed to using the robot. For example, some caregivers unfamiliar with the robot tended to use force while connecting the main body and the wearing unit and installing and removing the waste container from the main body for washing.

In contrast, RC, rather than MC, demonstrated lower muscular activities of ES, UT, and BB in the Front task. The front tasks are the primary tasks of EC. With MC, caregivers had to repeatedly change the positions of care receivers when they changed the diaper and washed the perineal area. These tasks can induce a physical load on the lower back, shoulders, and upper extremities. Budarick et al. [11] reported that turn-assist devices alleviate the force and movement components of the physical load on care receivers’ lower back and shoulders. However, CareBidet did not require patient turning. Therefore, in terms of physical load, CareBidet may be more beneficial for caregivers in Front tasks compared with the use of turn-assist devices.

In this study, MC and RC were compared for one round of EC. However, in an actual nursing home, MC is performed on an average of four to six times per elderly patient; hence, formal caregivers would experience a much greater physical load. On the other hand, tasks that use CareBidet are performed only once in a 24 h period. Therefore, CareBidet can support mitigating the workload for formal caregivers. However, the perception of caregivers about using EC robots should be improved to encourage the use the EC robots.

This study has four limitations. First, EC was not provided to actual living care receivers; hence, the results pertaining to muscle activity and subjective physical discomfort measured during MC and RC might have been distorted. Second, we did not confirm the interaction between the physical load and various factors that could affect the physical load of caregivers, such as anthropometric indices and demographic information; hence we will conduct a multivariate analysis in our future work. Third, the assessment was performed only on a small number of formal caregivers, so the study may be slightly biased. Fourth, the physical load of one round of MC and RC was compared without consideration of the number of times EC is provided in a day; hence, the nature of EC was not perfectly simulated. Future studies should recruit a larger caregiver sample and assess the physical load incurred when MC and RC are provided to care receivers with a consideration of the average number of times EC is provided in a day. Furthermore, the mental load can be assessed, and a model could be developed for the evaluation of the overall care burden of formal caregivers.

## 5. Conclusions

This study aimed to compare the physical load experienced by formal caregivers in the context of MC and RC through the measurement of muscular activity and subjective physical discomfort. We found that RC generated a greater physical load on the lower back and upper extremities during the preparation and post-care phases but markedly alleviated physical load during the actual performance of EC (Front tasks). These findings can provide a quantitative explanation of the features and effectiveness of a care robot in EC to stakeholders and thus serve as useful evidence for the implementation and development of care robots. Further, it provides foundational research data for the development of EC robots and is valuable in promoting the dissemination and popularization of care robots.

## Figures and Tables

**Table 1:** The specifications of CareBidet.

	Specifications
Product name	CareBidet
Dimension (mm)	Main body: 400 W × 400 H × 700 D
	Wearing unit: 2000
Weight (kg)	Main body: 25
	Wearing unit: 3
Capacity (cc)	Water bucket: 3000
	Excretion bucket: 5000
Voltage/Frequency	AC 220 V, 60 Hz
Materials	Silicon, etc.

## Data Availability

Not applicable.

## Appendix A

**Table A1:** The MC tasks.

Third Task Group	Second Task Group	First Task Group	Sub-Task
Preparation task	T.1. Preparation for excretion care	T.1.1. Bringing a care kit cart	T.1.1.1. Opening a door
			T.1.1.2. Bringing the cart to the side of a bed
			T.1.1.3. Fixing the cart
			T.1.1.4. Closing a door
		T.1.2. Disinfecting hands	T.1.2.1. Taking a hand sanitizer out of the cart
			T.1.2.2. Putting hand sanitizer on hands
		T.1.3. Wearing disposable gloves	T.1.3.1. Taking disposable gloves out of the cart
			T.1.3.2. Wearing disposable gloves
		T.1.4. Closing a privacy curtain	T.1.4.1. Closing a privacy curtain
		T.1.5. Folding a side rail down	T.1.5.1. Folding a side rail down
		T.1.6. Folding a blanket	T.1.6.1. Folding the blanket under the patient’s legs
Front task	T.2. Checking for excretion	T.2.1. Checking for excretion	T.2.1.1. Checking for excretion by smelling or touching diapers
	T.3. Preparation for excrement disposal	T.3.1. Putting a waterproof pad on a bed	T.3.1.1. Taking a waterproof pad out of the cart
			T.3.1.2. Putting the pad on a bed for a while
			T.3.1.3 Positioning the patient in a side-lying (lateral) position
			T.3.1.4 Holding the patient with one hand and putting the pad under the patient’s buttocks with the other hand
			T.3.1.5 Positioning the patient in a supine position
			T.3.1.6 Positioning the patient in a side-lying (lateral) position
			T.3.1.7 Putting the rest of the pad on a bed
			T.3.1.8 Positioning the patient in a supine Position
		T.3.2. Undressing the patient’s clothes	T.3.2.1. Raising the patient’s top to the waist
			T.3.2.2. Undressing the patient’s bottoms to the ankle
		T.3.3. Preparing wet wipes for cleaning	T.3.3.1. Taking wet wipes out of the cart
			T.3.3.2. Spraying EM on wet wipes
			T.3.3.3. Putting wet wipes on the cart
	T.4. Excrement disposal	T.4.1. Removing an inner diaper	T.4.1.1. Taking off the diaper sticker
			T.4.1.2. Raising the patient’s knees
			T.4.1.3. Folding the inner diaper and rolling the excrement
			T.4.1.4. Positioning the patient in a side-lying (lateral) position
			T.4.1.5. Wiping the patient’s buttocks with the inner diaper
			T.4.1.6. Rolling out the inner diaper
			T.4.1.7. Throwing away the inner diaper
		T.4.2. Preparing to wear a new outer diaper	T.4.2.1. Taking a new outer diaper out of the cart
			T.4.2.2. Putting a new outer diaper on the side of the patient
		T.4.3. Cleaning buttocks	T.4.3.1. Cleaning buttocks with the wet wipes
			T.4.3.2. Throwing away wet wipes
			T.4.3.3. Taking a dry towel out of the cart
			T.4.3.4. Removing moisture with a dry towel
			T.4.3.5. Putting the towel in the cart
		T.4.4. Removing an outer diaper	T.4.4.1. Rolling out the outer diaper under the patient’s buttocks
			T.4.4.2. Throwing away the outer diaper
	T.5. Wearing a new diaper	T.5.1. Wearing a new diaper	T.5.1.1. Putting a new outer diaper under the patient’s buttocks
			T.5.1.2. Taking a new inner diaper out of the cart
			T.5.1.3. Putting a new inner diaper on the outer diaper
			T.5.1.4. Folding the inner diaper’s waist and wing part
			T.5.1.5. Putting diapers close to the patient’s buttocks
			T.5.1.6. Positioning the patient in a supine position
			T.5.1.7. Raising the diapers
			T.5.1.8. Attaching stickers of the diapers
Post task	T.6. Completion of excretion care	T.6.1. Removing a waterproof pad	T.6.1.1. Folding the pad
			T.6.1.2. Positioning the patient in a side-lying (lateral) position
			T.6.1.3. Holding the patient with one hand and removing the pad under the patient’s buttocks with the other hand
			T.6.1.4. Positioning the patient in a supine position
			T.6.1.5. Positioning the patient in a side-lying (lateral) position
			T.6.1.6. Removing the rest of the pad
			T.6.1.7. Putting the pad in the cart
		T.6.2. Dressing the patient’s clothes	T.6.2.1. Dressing the patient’s bottom
			T.6.2.2. Dressing the patient’s top
		T.6.3. Covering the patient with a blanket	T.6.3.1. Covering the patient with a blanket
		T.6.4. Fixing a side rail	T.6.4.1. Fixing a side rail
		T.6.5. Opening a privacy curtain	T.6.5.1. Opening a privacy curtain
	T.7. Management and maintenance	T.7.1. Keeping a care kit cart	T.7.1.1. Unfastening the cart
			T.7.1.2. Opening a door
			T.7.1.3. Moving the cart
			T.7.1.4. Closing a door
			T.7.1.5. Keeping the cart

**Table A2:** The RC tasks.

Third Task Group	Second Task Group	First Task Group	Sub-Task
Preparation task	T.1. Preparation for excretion care	T.1.1. Bringing a care kit cart	T.1.1.1. Opening a door
			T.1.1.2. Bring the cart to the side of a bed
			T.1.1.3. Fixing the cart
			T.1.1.4. Closing a door
		T.1.2. Bringing a robot	T.1.2.1. Bring the robot to the side of a bed
			T.1.2.2. Bringing a wearing part and cover
			T.1.2.3. Fixing the robot
		T.1.3. Powering on the robot	T.1.3.1. Plugging the cord
			T.1.3.2. Powering on the robot
		T.1.4. Opening the robot’s body	T.1.4.1. Opening the robot’s body
		T.1.5. Filling a cleaning water bottle	T.1.5.1. Taking out the bottle from the robot’s body
			T.1.5.2. Moving to the bathroom with the bottle
			T.1.5.3. Opening the lid of the bottle
			T.1.5.4. Filling the bottle
			T.1.5.5. Closing the lid of the bottle
			T.1.5.6. Moving to the room with the bottle
			T.1.5.7. Installing the bottle on the robot’s body
		T.1.6. Connecting a wearing part to the robot body	T.1.6.1. Installing the wearing part on the slide board of the robot’s body
			T.1.6.2. Connecting a cable of the wearing part to the robot’s body
			T.1.6.3. Installing a detachable cushion on the wearing part
		T.1.7. Closing the robot’s body	T.1.7.1. Closing the robot’s body
		T.1.8. Disinfecting hands	T.1.8.1. Taking a hand sanitizer out of the cart
			T.1.8.2. Putting hand sanitizer on hands
		T.1.9. Wearing disposable gloves	T.1.9.1. Taking disposable gloves out of the cart
			T.1.9.2. Wearing disposable gloves
		T.1.10. Closing a privacy curtain	T.1.10.1. Closing a privacy curtain
		T.1.11. Folding a side rail down	T.1.11.1. Folding a side rail down
		T.1.12. Folding a blanket	T.1.12.1. Folding a blanket
Front task	T.2. Preparation for wearing a care robot	T.2.1. Covering the wearing part	T.2.1.1. Spreading the abdominal band and waistband on the cover
			T.2.1.2. Opening the top zipper and bottom zipper to the sewing line
			T.2.1.3. Putting the wearing part in the bottom pocket of the cover
		T.2.2. Fastening the cover with the wearing part	T.2.2.1. Closing the bottom zipper
			T.2.2.2. Fastening buttons between the wearing part and the inside of the cover
		T.2.3. Preparing the wearing part	T.2.3.1. Rolling the waistband
			T.2.3.2. Putting the wearing part on the side of the patient
		T.2.4. Undressing the patient’s clothes	T.2.4.1. Raising the patient’s top to the waist
			T.2.4.2. Undressing the patient’s bottoms
	T.3. Wearing a care robot	T.3.1. Removing an inner diaper	T.3.1.1. Taking off the diaper sticker
			T.3.1.2. Raising the patient’s knees
			T.3.1.3. Rolling out the inner diaper
		T.3.2. Removing an outer diaper	T.3.2.1. Rolling out the outer diaper
			T.3.2.2. Throwing away the diapers
		T.3.3. Attaching the wearing part to the patient’s body	T.3.3.1. Push the waistband of the cover connected to the wearing part down the patient’s waist
			T.3.3.2. Attaching the wearing part to the patient’s buttocks (horizontally)
		T.3.4. Wearing the wearing part	T.3.4.1. Putting one hand between the patient’s thighs and holding the front of the wearing part
			T.3.4.2. Positioning the patient in a supine position
		T.3.5. Adjusting the wearing part	T.3.5.1. Grabbing the waistband of the cover
			T.3.5.2. Pulling the waistband of the cover from side to side to ensure that the part is positioned in the center of the wearing part
		T.3.6. Completing wearing the wearing part	T.3.6.1. Covering the patient’s abdomen with a waistband
			T.3.6.2. Installing a gender module cup on the wearing part
			T.3.6.3. Pulling up the abdominal band of the cover and closing the top zipper
			T.3.6.4. Spreading the proof band of the cover
			T.3.6.5. Spreading the patient’s buttocks
			T.3.6.6. Pressing the operation button of the robot body
	T.4. Completion of wearing a care robot	T.4.1. Dressing the patient’s clothes	T.4.1.1. Dressing the patient’s bottom
			T.4.1.2. Dressing the patient’s top
		T.4.2. Covering the patient with a blanket	T.4.2.1. Covering the patient with a blanket
		T.4.3. Fixing a side rail	T.4.3.1. Fixing a side rail
		T.4.4. Opening a privacy curtain	T.4.4.1. Opening a privacy curtain
	T.5. Preparation for detaching a care robot	T.5.1. Closing a privacy curtain	T.5.1.1. Closing a privacy curtain
		T.5.2. Folding a side rail down	T.5.2.1. Folding a side rail down
		T.5.3. Folding a blanket	T.5.3.1. Folding a blanket
		T.5.4. Undressing the patient’s clothes	T.5.4.1. Raising the patient’s top to the waist
			T.5.4.2. Undressing the patient’s bottoms
	T.6. Detaching a care robot	T.6.1. Preparing to remove the wearing part	T.6.1.1. Opening the robot’s body
			T.6.1.2. Installing a holder of the wearing part
			T.6.1.3. Pressing the stop button of the robot body
		T.6.2. Removing the wearing part	T.6.2.1. Opening the top zipper of the cover
			T.6.2.2. Removing the gender module cup
			T.6.2.3. Opening the bottom zipper of the cover
			T.6.2.4. Unfastening the fastening buttons
			T.6.2.5. Removing the wearing part
			T.6.2.6. Hanging the wearing part on the holder
			T.6.2.7. Fastening the wearing part with a belt on the holder
			T.6.2.8. Hanging the hose on the hose rack of the robot’s body
		T.6.3. Preparing to wear a new outer diaper	T.6.3.1. Taking a new outer diaper out of the cart
			T.6.3.2. Putting a new outer diaper on the side of the patient
		T.6.4. Removing the cover	T.6.4.1. Opening the abdominal band and waistband of the cover
			T.6.4.2. Rolling the waistband and pushing it inside the patient’s waist
			T.6.4.3. Turning the patient’s neck and grabbing their knees and shoulders
			T.6.4.4. Positioning the patient in a side-lying (lateral) position
			T.6.4.5. Removing the cover and putting the cover in the cart
		T.6.5. Wearing a new diaper	T.6.5.1. Putting a new outer diaper under the patient’s buttocks
			T.6.5.2. Taking a new inner diaper out of the cart
			T.6.5.3. Putting a new inner diaper on the outer diaper
			T.6.5.4. Folding the inner diaper’s waist and wing part
			T.6.5.5. Putting diapers close to the patient’s buttocks
			T.6.5.6. Positioning the patient in a supine position
			T.6.5.7. Raising the diapers
			T.6.5.8. Attaching stickers of the diapers
Post task	T.7. Completion of excretion	T.7.1. Dressing the patient’s clothes	T.7.1.1. Dressing the patient’s bottom
			T.7.1.2. Dressing the patient’s top
		T.7.2. Covering the patient with a blanket	T.7.2.1. Covering the patient with a blanket
		T.7.3. Fixing a side rail	T.7.3.1. Fixing a side rail
		T.7.4. Opening a privacy curtain	T.7.4.1. Opening a privacy curtain
		T.7.5. Powering off the robot	T.7.5.1. Powering off the robot
			T.7.5.2. Unplugging the cord
	T.8. Management and maintenance of a care robot	T.8.1. Disconnecting an excreta collection container and hose	T.8.1.1. Disconnecting an excreta collection container and hose
			T.8.1.2. Disconnecting an excreta collection container and a cleaning water bottle from the robot body
		T.8.2. Cleaning the excreta collection container and the cleaning water bottle	T.8.2.1. Moving to the bathroom with the excreta collection container and the cleaning water bottle
			T.8.2.2. Opening the lid of the excreta collection container
			T.8.2.3. Throwing the contents away in the toilet
			T.8.2.4. Flushing the toilet
			T.8.2.5. Opening the container by unlocking the airtight cover
			T.8.2.6. Removing the guard of the container
			T.8.2.7. Washing the container
			T.8.2.8. Removing moisture from the container
			T.8.2.9. Closing the lid of the container
			T.8.2.10. Opening the lid of the bottle
			T.8.2.11. Washing the bottle
			T.8.2.12. Removing moisture from the bottle
			T.8.2.13. Closing the lid of the bottle
			T.8.2.14. Moving to the room with the excreta collection container and the cleaning water bottle
			T.8.2.15. Installing the excreta collection container and the cleaning water bottle on the robot body
		T.8.3. Closing the robot’s body	T.8.3.1. Keeping the supplies in the robot’s body
			T.8.3.2. Closing the robot’s body
		T.8.4. Keeping the robot	T.8.4.1. Unfastening the robot
			T.8.4.2. Keeping the robot
			T.8.4.3. Keeping the wearing part and cover
	T.9. Management and maintenance	T.9.1. Keeping the cart	T.9.1.1. Unfastening the cart
			T.9.1.2. Opening a door
			T.9.1.3. Moving the cart
			T.9.1.4. Closing a door
			T.9.1.5. Keeping the cart
